# Radioactive Beams in Particle Therapy: Past, Present, and Future

**DOI:** 10.3389/fphy.2020.00326

**Published:** 2020-08-28

**Authors:** Marco Durante, Katia Parodi

**Affiliations:** 1 Biophysics Department, GSI Helmholtzzentrum für Schwerionenforschung, Darmstadt, Germany; 2 Institute of Condensed Matter Physics, Technische Universität Darmstadt, Darmstadt, Germany; 3 Department of Experimental Physics—Medical Physics, Ludwig-Maximilians-Universität München, Munich, Germany

**Keywords:** particle therapy, radioactive ion beams, carbon ions, oxygen ions, PET

## Abstract

Heavy ion therapy can deliver high doses with high precision. However, image guidance is needed to reduce range uncertainty. Radioactive ions are potentially ideal projectiles for radiotherapy because their decay can be used to visualize the beam. Positron-emitting ions that can be visualized with PET imaging were already studied for therapy application during the pilot therapy project at the Lawrence Berkeley Laboratory, and later within the EULIMA EU project, the GSI therapy trial in Germany, MEDICIS at CERN, and at HIMAC in Japan. The results show that radioactive ion beams provide a large improvement in image quality and signal-to-noise ratio compared to stable ions. The main hindrance toward a clinical use of radioactive ions is their challenging production and the low intensities of the beams. New research projects are ongoing in Europe and Japan to assess the advantages of radioactive ion beams for therapy, to develop new detectors, and to build sources of radioactive ions for medical synchrotrons.

## Introduction

Currently, ~50% of cancer patients in Europe experience radiotherapy, generally by X-rays, as part of their treatment [1]. In recent years, photon radiotherapy has greatly improved its accuracy and safety thanks to image guidance (IGRT) [2]. However, charged particle therapy (CPT) with protons and light ions is rapidly growing all over the world, particularly in Europe [3]. In fact, thanks to the favorable depth-dose distribution, more normal tissue is spared with CPT compared to conventional radiotherapy with X-rays in virtually all sites, leading to high success/toxicity ratios [4]. Using ions heavier than protons, generally carbon ions, the physics advantages are added to the radiobiological properties, being stopping (high-LET) ions in the tumor region more effective than X-rays or protons for cell killing, while in the normal tissue, fast (low-LET) ions induce a toxicity comparable to sparsely ionizing radiation [5]. The experience at the National Institute of Radiological Sciences (NIRS) in Chiba (Japan) [6] and in the European centers [7] demonstrates that the radiobiological and physical rationale is actually translated in improved clinical results for several indications [8].

**Figure 1:**
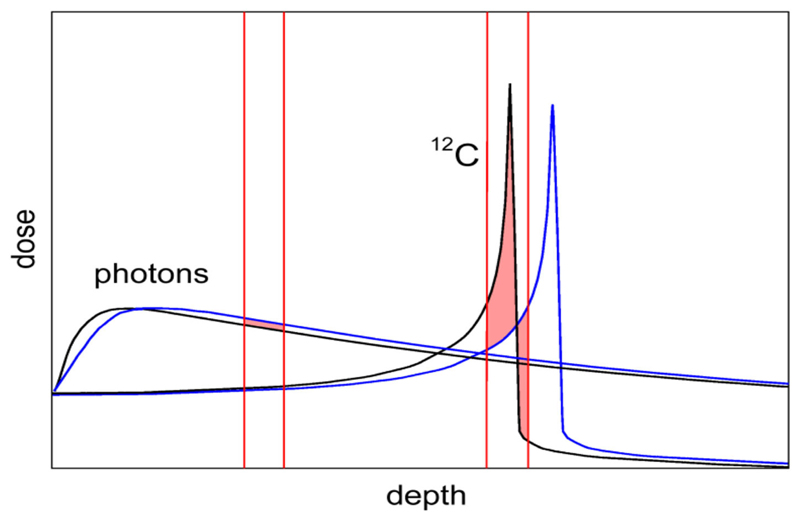
Comparison of depth-dose distribution for heavy ions (^12^C) and photons (X-rays). The Bragg peak gives the physical advantages of CPT. However, the figure shows that a small range shift caused, e.g., by a tissue inhomogeneity has a small impact on the X-ray curve, but in CPT it can significantly shift the Bragg peak from the target into a sensitive organ surrounding the tumor.

Yet, CPT remains controversial [9]. The first reason is the higher cost of the CPT facilities [10], especially the expensive heavy ion centers. Even if the cost is still much higher for particle therapy centers compared to linacs for X-rays, it is declining, mostly thanks to superconductive technologies now employed for the construction of the accelerators (cyclotrons, synchro-cyclotrons, or synchrotrons) [11, 12]. However, CPT is also limited in what should be the main advantage, i.e., the high precision made possible by the Bragg peak. CPT is indeed less robust than conventional radiotherapy because of considerable uncertainty on the particle range and poor image guidance [13]. While the lateral penumbra is shallower for protons than for X-rays, making the proton plans robust for misalignments in the direction orthogonal to the beam direction [14], for heavy ions, characterized by sharp dose gradients in all directions and very high doses in the distal ends, range uncertainty is the main physics limitation. Image guidance is essential for CPT, even more so than for X-rays, because a shift in the Bragg peak has a much larger impact on the dose than for photons (Figure 1). For moving targets this also occurs through the interplay effect, causing underdosage to part of the target [15]. In-room CT and cone-beam CT are emerging as the two image guidance methods of choice for CPT, but IGRT using X-rays is more accurate and robust [16] and is quickly improving thanks to the recent introduction of online magnetic resonance imaging (MRI) [17, 18]. Clinically, a substantial margin is added in CPT to the prescribed range in order to ensure tumor coverage, e.g., in proton therapy, this range margin is on the order of 3.5% of the prescribed range [19]. Wide margins jeopardize one of the main advantages of the Bragg peak: the steep dose gradients and the potential high accuracy and precision [20].

To tackle the range uncertainty problem, several methods for range verification have been developed. Imaging in radiology very often uses radioactive tracers, and it was indeed proposed already long ago [21] that radioactive ion beams (RIB) have the potential for simultaneous treatment and beam visualization, similar to theranostics with radioisotopes [22]. We will first describe the current methods for heavy ion beam visualization, and then the past experience is using RIB in cancer therapy. We will then argue that the current efforts for high-intensity accelerators can lead to a more effective use of RIB in therapy, pending experimental proof of the clinical advantages.

## Range Verification in Particle Therapy

Even if image guidance is less common in CPT compared to conventional radiotherapy, the physics of charged particles offers unique opportunities for *in vivo* range verification. In proton therapy, there is an increasing use of prompt γ-ray detectors that measure the emission of photons by nuclear reactions and their fast decay shortly before the Bragg peak [23]. The method has been tested also for high-energy C-ions in phantoms [24, 25]. Several other methods have been proposed, such as ionoacoustic measurements [26] or mixed beams [27]. For C-ions, it is also possible to measure secondary charged particles, such as protons emitted at large angles [28, 29]. A combination of different methods is under study for animal irradiators [30] and in clinical settings [31, 32]. Reviews of different methods for *in vivo* range verification can be found in Refs. [33–36].

**Figure 2:**
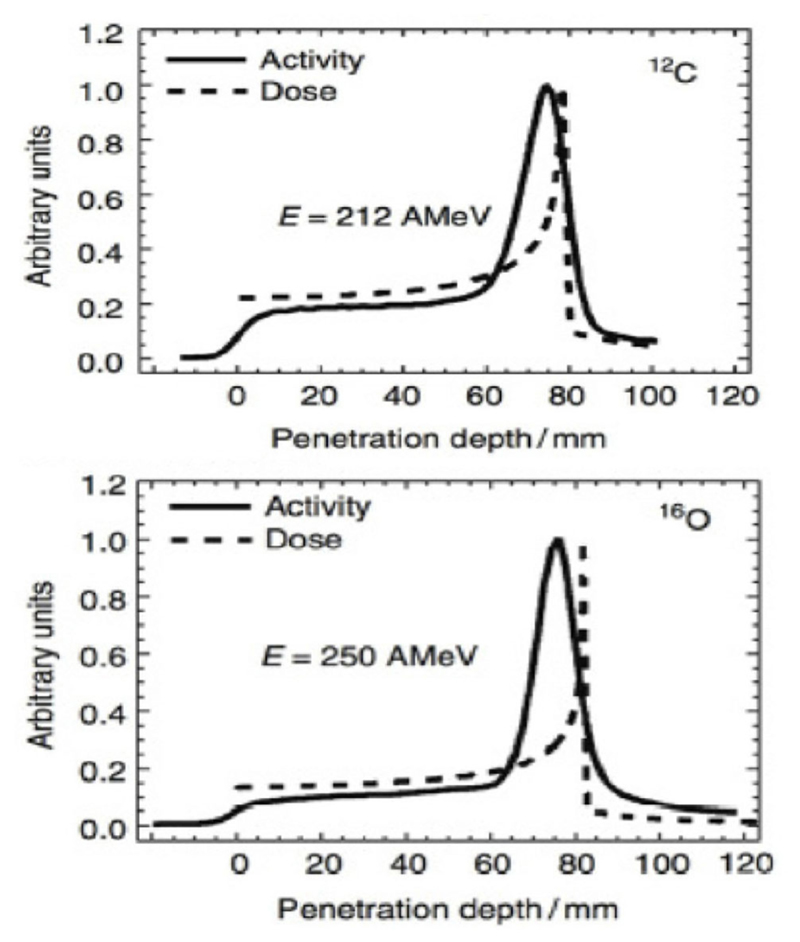
Measured PET activity in PMMA phantoms irradiated at GSI with carbon or oxygen ions, showing the shift between activity and dose peak (measurements from Ref. [39]).

The range verification method that has been tested most extensively in clinical practice is positron emission tomography (PET) [37]. PET is a well-known diagnostic imaging method, based on the detection of the two 511 keV photons emitted by annihilation of a positron with an electron in the media. Unlike conventional diagnostic imaging [38], currently PET in particle therapy exploits β^+^-emitting isotopes produced by the particle beam in the patient’s body by nuclear fragmentation [37]. In proton therapy, only target fragments can be used for imaging, while in heavy ion therapy, the projectile fragments provide a large part of the signal with better correlation to the dose. A list of typical radionuclides produced by target fragmentation in proton therapy or potential projectile fragments is provided in Table 1. The radioactive projectile fragments provide a peak in the activity that is not observed in proton therapy (Figure 2) [40]. However, the activity peak invariably occurs upstream of the Bragg peak, because the light isotopes of the projectile have shorter range at the same velocity of the primary ion [13, 39]. Online PET was used for the first time clinically during the ^12^C-ion pilot therapy project at GSI, Darmstadt, until 2008 [41], and a number of CPT centers are currently using PET for beam verification [32, 42–44], usually offline.

**Figure 3:**
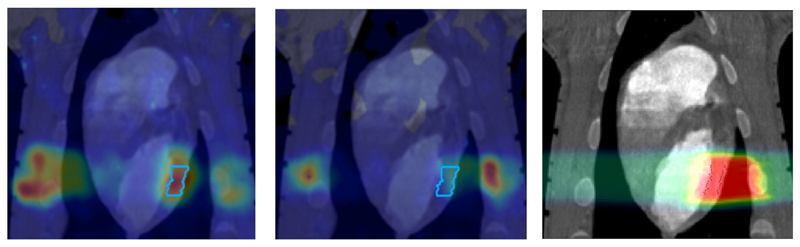
PET images of a pig heart treated with ^12^C-ions. The ventricular target is drawn in the treatment planning image overlaid to the CT **(Right)**. Online PET image **(Left)**was acquired during the treatment at GSI, while the offline (center) was registered 20 min after the treatment. PET imaging obtained with the online PET camera at GSI, courtesy of Helmholtzzentrum Dresden (HZDR); details in Ref. [47].

However, PET in C-ion therapy remains marginal and not really able to reduce range uncertainty as desired. The half-life of the most abundant induced radionuclides is too long for instantaneous feedback (Table 1), and the short-lived radionuclides are produced at a very low rate and exhibit a long positron range [45]. The measured activity is not directly correlated to the Bragg curve in phantoms (Figure 2), and the situation is worsened *in vivo* by the biological washout [43, 46]. An example comes from recent experiments on heavy-ion treatment of heart arrhythmia in a swine model, where online PET was used for range verification of a C-ion beam [47]. In Figure 3, we compare online to offline PET in a pig heart ventricular target irradiated with ^12^C-ions. After 20 min, only the signal in the ribs is still visible in PET. The lack of a direct correlation with the dose (Figure 2) and the washout (Figure 3) makes resorting to Monte Carlo (MC) simulations [40] or other analytical calculations [48] currently unavoidable for data analysis. Furthermore, the activity is time-dependent according to the half-lives of the isotopes (Table 1) and the efficiency of the detector system in measuring the activity distribution. All these corrections currently limit the accuracy of PET-based range verification to about 2−5 mm [33, 42, 49].

## RIB in Radiotherapy

The rationale for using RIB in therapy has looked in two directions. On one side, it was assumed that the radioactive decay can increase the dose in the target. This was similar to the rationale for using antiprotons [50] or pions [51] for therapy. Among radioactive isotopes, ^9^C attracted attention because of its β-delayed decay in low-energy, densely ionizing particles [52]. However, despite some successful *in vitro* experiments [53], these approaches have been abandoned. The energy released by nuclear reaction in the target is indeed in the order of the nuclear shell energies, and such energy is always very small compared to the electromagnetic energy loss of the particle in the tumor. In fact, simulations show that the putative increase due to nuclear reactions in the target is negligible [54].

On the other hand, RIB can be used for image-guided particle therapy. In fact, the best way to increase the signal intensity in online PET would be the use of β^+^ emitters for treatment. Using RIB, every primary ion will decay, essentially only at the end of the range, with the decay time always much longer than the travel time in the accelerator and in the patient’s body. RIB would improve the count rate ~10 × [55], reduce the shift between measured activity and dose (Figure 2), and mitigate the washout blur of the image (Figure 3) with short-lived isotopes and in-beam acquisition. Heavy ion therapy is nowadays only performed using carbon ions, because with heavier ions, the toxicity in normal tissues can be unacceptable. The Heidelberg Ion Therapy (HIT) center is currently planning to use oxygen ions for radioresistant tumors, and therefore looking at Table 1, one should consider isotopes of C, N, and O as potential projectiles in RIB therapy.

The idea of using RIB in therapy is certainly not new, as the potential advantage in terms of improved precision and accuracy was clear since the beginning of CPT. Below, we will describe past efforts in this direction.

### Lawrence Berkeley Laboratory

**Figure 4:**
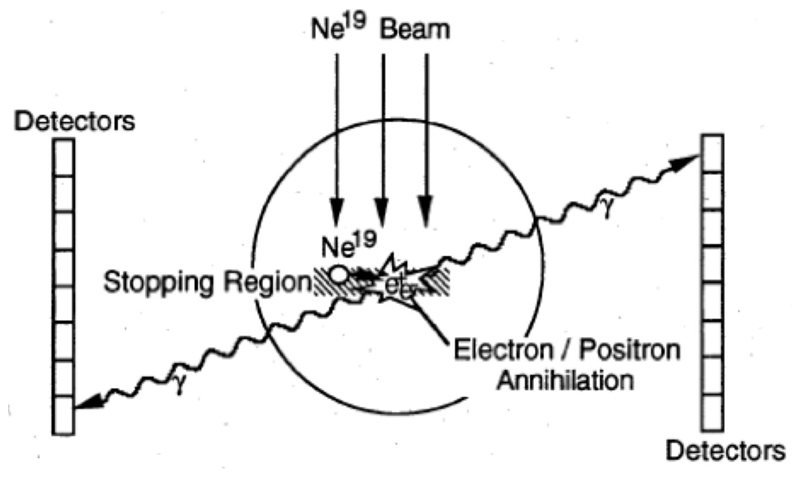
PEBA detector developed at LBL for the visualization of ^19^Ne ion range. Figure from Ref. [58].

Cancer therapy using ions heavier than protons was first tested in the pilot project of the Lawrence Berkeley Laboratory (LBL) in USA led by Cornelius A. Tobias. The project started in 1975 and used He, C, Ne, Si, and Ar ions, treating 1,314 patients until the shutdown of the Bevalac accelerator in 1992 [56, 57]. The uncertainty in predicting the correct range of heavy ions from the CT images, produced by X-rays, was soon clear and the LBL physicists explored the possibility of using RIB for range verification [58]. The LBL studies focused on ^19^Ne (Table 1) and built a modified PET detector (PEBA) consisting of two arrays of 64 BiGe scintillators in an 8 × 8 matrix arrangement, which are separated by a distance of ~1 m (Figure 4). PEBA was already able to demonstrate an accuracy of ~1 mm in range determination in phantoms [21].

### Eulima

The European Light Ion Medical Accelerator (EULIMA) project was funded by EU within the 2nd Framework Program in 1989. The project was led by the cyclotron laboratory in Nice, which was already active in proton therapy for eye treatment [59]. The concerted action studied the feasibility of a hospital-based light ion (2 ≤ *z* ≤ 10) accelerator facility for the treatment of a large number of cancer patients in Europe. The project explored the idea of using a superconducting cyclotron, based on the experience in Nice, and carefully analyzed the option of irradiating the patients with radioactive isotopes of carbon, oxygen, or neon. Cyclotrons have the advantage of high intensity and simplicity of operation. However, superconducting cyclotrons for ions as heavy as carbon requires an intense R&D for magnetic field shaping and high voltage. Synchrotrons are instead flexible machines, energy can be rapidly changed, different ion species can be accelerated, and they are a well-established technology. For these reasons, the EULIMA feasibility study recommended using synchrotrons for heavyion therapy [60], and indeed all European ion beam centers are currently using synchrotrons. IBA, the leading company in cyclotrons for proton therapy, is still working on the idea of the superconducting cyclotron for carbon ions (C400) [61], in collaboration with GANIL at Caen (France), but the project is still ongoing.

## GSI

**Figure 5:**
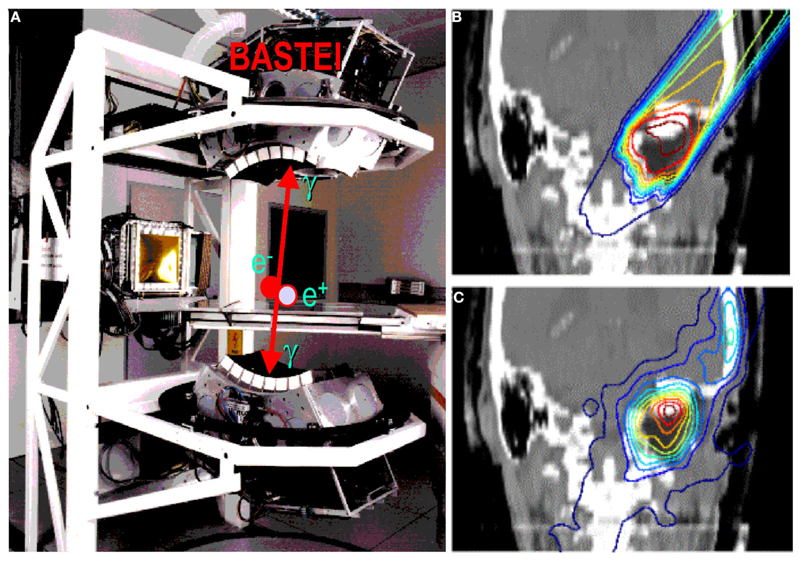
**(A)** The PET camera (without housing) installed at the GSI treatment room (cave M) and used during the pilot project, as shown in the clinical case in **(B)** (prescribed dose according to treatment planning) and **(C)** (measured activity distribution, modified by the washout).

The GSI Helmholtz Center for Heavy Ion Research in Darmstadt (Germany) treated the first patient in Europe with ions heavier than protons—carbon ions. The program was led by Gerhard Kraft and treated 440 patients with ^12^C-ions between 1997 and 2008 [5, 62]. As noted in section Range Verification in Particle Therapy, the pilot project at GSI used for the first time PET online to verify the dose delivery (Figure 5). The group from Helmholtz Center Dresden that worked on the PET system also measured RIB, produced at the GSI fragment separator (FRS) [63]. They used ^15^O, ^17^F, and ^19^Ne for testing the PET camera [64]. All patients in the pilot project were, however, treated with stable ^12^C ions and PET images exploited mostly the ^11^C projectile fragment produced by nuclear fragmentation. As shown in Figure 3, the same PET camera was recently used for irradiation of AV nodes and ventricles in swine hearts at GSI [47]. Radiotherapy for treatment of heart arrhythmia is considered a very promising non-invasive alternative to catheter ablation [65], and recent results with stereotactic radiosurgery for ventricular arrhythmia are very encouraging [66]. Charged particles are potentially much more effective for these kinds of treatment [67] because they require single high doses, and with X-rays, this can cause severe toxicity in the normal heart and other surrounding critical structures such as esophagus and lungs. However, the cardiac targets are small and rapidly moving, and therefore PET imaging plays a very important role for applications of heavy ions in non-cancer diseases. The first patient with ventricular arrhythmia has been treated with protons at CNAO (Pavia, Italy) in December 2019 [68].

## HIMAC

**Figure 6:**
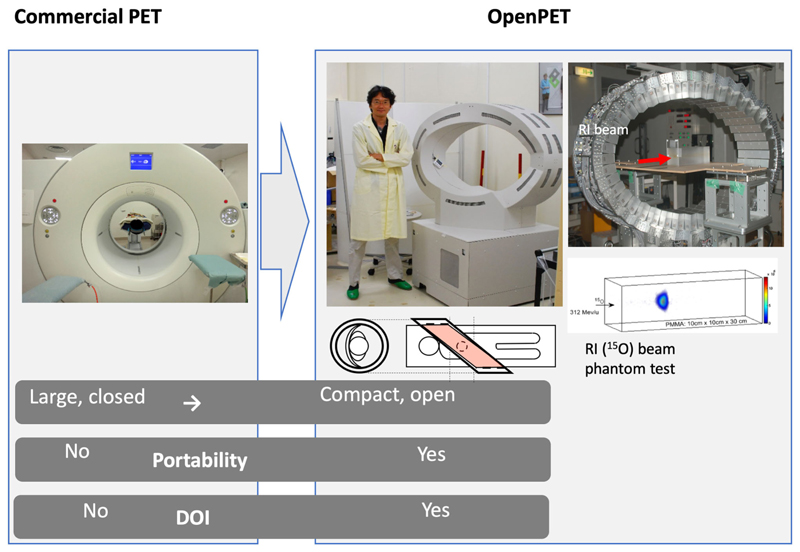
The OpenPET imaging detector developed at NIRS in the Taiga Yamaya laboratory, along with images of a ^15^O beam in a plastic target. Image from https://www.nirs.qst.go.jp/usr/medical-imaging/imaging-physics/index-en.html, reproduced with permission.

Certainly, the accelerator facility that has the longest history and success in RIB production and testing for cancer therapy is the HIMAC at NIRS in Chiba, Japan. Following the LBL pilot project, NIRS was the first center to treat patients with ions heavier than protons, specifically carbon ions. NIRS used the flexible and reliable HIMAC synchrotron for patient treatments and research [69], and most of the patients treated worldwide with C-ions were actually irradiated at HIMAC [6]. In over 20 years of clinical operation, NIRS has demonstrated excellent results in many tumor sites with acceptable toxicity, very often in hypofractionation [70]. NIRS has always invested in research and development in heavy ion therapy and has been studying RIB for therapy for 20 years [71]. Considering the low RIB intensity (see section RIB Production), NIRS physicists were trying to use the RIB beam at low intensity as a probe before application of the stable carbon therapeutic beam. The Yamaya laboratory at NIRS has developed a new concept of open-PET [72–74] (Figure 6) to visualize the beam and has applied the system to study the washout of radionuclides in animal targets [75, 76]. Optical beam imaging has also been recently used to visualize RIB at HIMAC [77]. The HIMAC studies demonstrate that RIB have similar radiobiological properties as stable isotopes of the same atomic number but produce far better quality images for range verification, with 5–11-fold improvements in the PET signal/noise ratio [78].

## RIB Production

**Figure 7:**
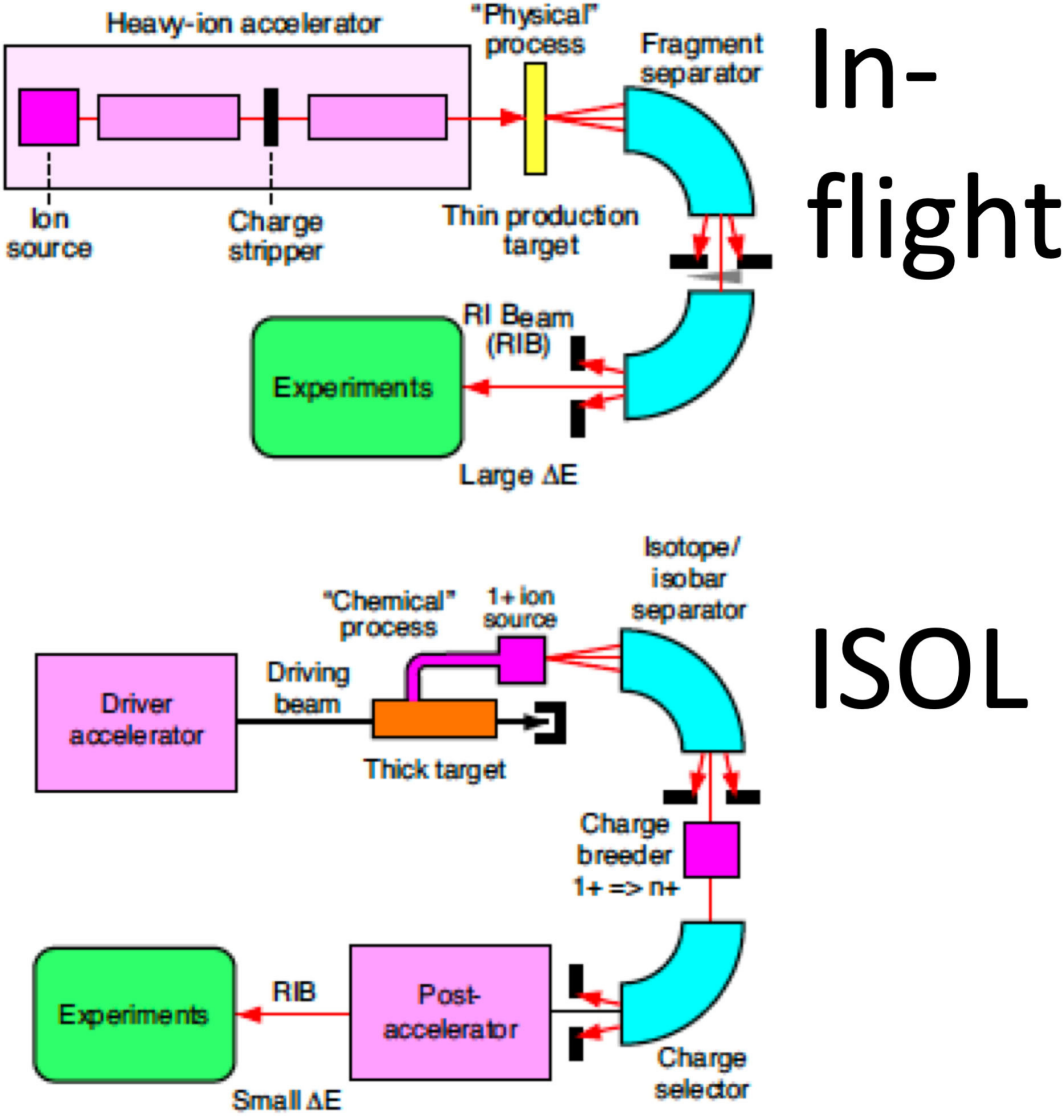
Schematic drawing of the in-flight and ISOL methods for RIB production.

The main hindrance to the full exploitation of RIB in cancer therapy is the low intensity. RIB are a very important modern topic in nuclear physics, as they allow to study the properties of nuclear matter far from the stability curve [79]. To produce RIB, two techniques are used at particle accelerators: Isotope-online (ISOL) and in-flight [80, 81] (Figure 7). ISOL is based on light-ion (usually ^1^H or ^2^H)-induced spallation or fission of thick targets (Ta or U). The radioactive fragments are extracted from the thick target through thermal diffusion at high temperature, effused to an ion source to become singly charged ions and finally accelerated toward a target. RIB production for therapy has so far used the in-flight technique, where RIB are obtained by fragmentation of the stable primary beam in thin targets (usually in C or Be). The reaction fragments, ejected in the forward direction with almost the same speed as that of the incident beam, are magnetically separated and then transferred to the experimental vault. The RIB (*A, N*-1) intensity is therefore determined by the fragmentation cross section of the primary beam (*A, N*). As shown in Table 2, the production cross section for light ions at high energy is ~45 mb per one nucleon, and decreases an order of magnitude for every further nucleon. Beam intensity is consequently reduced to < 10^−2^ for *N*-1 isotopes and 10^−3^ for *N*-2 isotopes. At HIMAC, beams of ^11^C were produced with intensities ranging 10^5^–10^6^ pps [77, 82], still too low for a therapeutic C-ion treatment that requires 10^8^–10^9^ pps [5, 13].

An additional problem in the in-flight technique is the large momentum spread. This spread causes a shift between the Bragg peak and activity peak for RIB [83]. Even if this shift is smaller than the one observed using stable ions for treatment and projectile fragments for PET imaging (Figure 2), it increases with the momentum acceptance. Recent measurements at HIMAC shows that for ^11^C, the shift is around 2 mm at 5% acceptance and is reduced to 0.1 mm at 0.5% momentum acceptance [84]. Momentum spreads can therefore translate in significant range spreads at the site of stopping (Table 2).

In-flight production of RIB would be impractical in current medical synchrotrons. Already at LBL, it was hypothesized to produce the RIB at low energy and then inject them in the high-energy medical accelerator [21]. The idea is to build a small cyclotron that can produce low-energy RIB with an ISOL system, and these ions are then injected in conventional synchrotrons. A source using low-energy electron beams for the production of ^11^C has been designed and produced at HIMAC [85]. Within the MEDICIS-Promed project [86], CERN has proposed a charge breeding scheme based on an Electron Beam Ion Source for beam preparation of a radioactive ^11^C beam [87]. The charge breeder is coupled to a medical synchrotron currently used for ^12^C-ion therapy (such as MedAustron) to treat patients with ^11^C using the same beam delivery devices of conventional heavy-ion therapy [88].

## BARB

**Figure 8:**
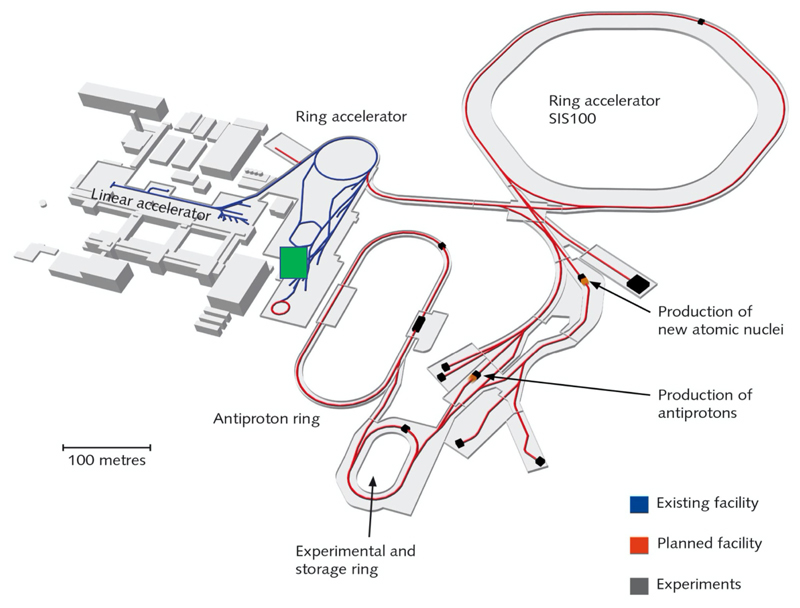
Layout of the FAIR facility under construction in Darmstadt. The blue lines represent the current accelerator, including the SIS18 ring, and the red lines represent the new beam pipes under construction. BARB will work on the SIS18 and its exit beamline in Cave M (indicated by a green square in the map), previously used for the therapy project.

GSI-treated cancer patients with ^12^C-ions accelerated at SIS, a 18 Tm synchrotron where the FRS has been used for many nuclear physics experiments [63]. SIS18 will be the injector of a new accelerator at 100 Tm, currently under construction for the Facility for Anti-protons and Ion Research (FAIR) [89] (Figure 8). A new FRS (super-FRS) will be built at SIS100 [90], to accommodate the ambitious physics program of the NuSTAR collaboration [91]. In addition to the nuclear physics program, FAIR also includes a large applied physics program (APPA) in atomic physics, plasma physics, materials research, and biophysics [92]. The biophysics program at FAIR aims at exploiting the intensity and energy upgrades for therapy and space radiation protection research [93]. While SIS100 is under construction, the FAIR-phase-0 is already ongoing with the main goal of increasing the intensity by a factor of × 10,000 compared to the current values [94].

The intensity upgrade at SIS18 can be exploited to test RIB therapy in the same Cave M (Figure 5) where the pilot project was performed. The project Biomedical Applications of Radioactive ion Beams (BARB) (www.gsi.de/BARB) aims at testing ^10,11^C and ^14,15^O for simultaneous treatment and imaging at FAIR, with the goal of reaching sub-mm precision in range verification and to demonstrate the potential of RIB therapy in an animal model. BARB is funded by EU within the 2019 ERC Advanced Grant call and is a 5-year project starting in late 2020.

## FRS at FAIR

The radioactive ions of interest will be produced by fragmentation (one- or two-neutron removal, respectively) of relativistic primary beams (^12^C, ^16^O) in reaction targets (Be, C) placed at the entrance of the SIS18 FRS and separated in-flight [63]. As discussed in section RIB Production, the intensity of the RIB depends on the primary beam current, on the fragmentation cross-sections, and on the transport properties. Table 2 gives the result of a Monte Carlo simulation with the GSI code MOCADI [95] using the intensities expected at SIS18 in FAIR-phase-0. The experimental activity in this task will focus on optimization of the accelerator parameters to reach the maximum intensities. The intensity in Cave M must be verified experimentally and critically depends on the size of the beam to be used for dosimetry and pre-clinical experiments in a mouse model. The MOCADI simulation indicates a range straggling σ/R ~ 2.5% for both light ions in the energy range of interest for therapy. The range straggling is a direct consequence of the momentum spread discussed in section RIB Production. Range straggling will therefore be carefully assessed during BARB in order to reach sub-millimeter precisions. It is also possible to apply methods to produce mono-energetic, pencil-like secondary beams for therapy, e.g., using the energy-focusing method that was developed at the FRS [96].

### Hybrid Detector

**Figure 9:**
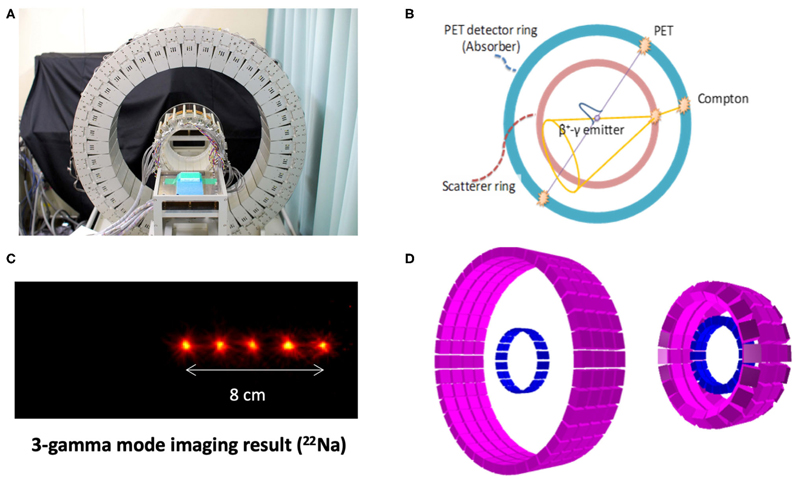
WGI prototype at NIRS **(A)** for combined PET and Compton imaging **(B)**, along with its Compton imaging performance for the measurement of ^22^Na sources, emitting a positron, and a 1,274 keV photon **(C)**. Corresponding simulation model **(D)** for optimization of the design [left: same design as **(A)**, right: modified design for improved Compton efficiency] (Source: NIRS and LMU).

The second innovative aspect of BARB is the use of a new γ-PET detector that will be designed and built at LMU. Cave M is equipped with an online PET (Figure 5), but even online PET can only register in-between the synchrotron beam spills, because the signal is obscured by the large prompt γ-ray signal during the irradiation [72, 97]. An improved detector should be able to exploit the prompt γ-ray emission [23] during beam extraction, in addition to the PET acquisition (and concomitant third-γ emission in case of ^10^C and ^14^O) in-between the synchrotron spills. BARB will build a hybrid detector concept aiming to exploit both the prompt γ-rays emitted in nuclear interactions during the beam-on time of the synchrotron pulsed delivery, and the delayed emission of the (γ-)β^+^-emitting primary beam (superimposed to a minor contribution of positron emitting projectile and target fragments) in the beam pauses [37]. The new detector concept will be based on an advanced version of the γ-PET design originally proposed at LMU [98] and further developed in the framework of the International Open Laboratory and International Research Initiative between LMU and NIRS (Figure 9). The focus of these joint NIRS-LMU efforts has been on the imaging of nuclear medicine tracers that undergo β^+^-decay with simultaneous emission of a third prompt photon from the excited daughter nucleus, thus making it possible to achieve improved imaging performances by the intersection of the annihilation photons’ line of response (LOR) and the third photon Compton cone [98]. A promising proof of concept of this so-called whole gamma imaging (WGI) [99] approach could be already demonstrated at NIRS in a mouse using ^89^Zr, which has a β^+^ and electron capture decay in ^89m^Y with a half-life of 78 h. ^89m^Y finally decays into the stable ^89^Y by an emission of 909 keV γ-ray and a half-life of 15.7 s [100]. Hybrid PET, Compton, and Compton-PET imaging were obtained relying on the addition of a scatterer ring (94 mm diameter) made of GAGG scintillator crystals inside a full-size (660 mm diameter) PET scanner with depth-of-interaction Zr-doped GSO scintillator detectors already available at NIRS [101, 102]. While nuclear medicine tracer imaging is limited to single-γ energies up to ~1 MeV, the energy of interest of prompt-γ typically lies in the 3–8 MeV interval and is a priori unknown. Hence, recent research at LMU has focused on design studies aiming to upgrade the NIRS detector in terms of enhanced efficiency of Compton imaging at these higher PG energies, without compromising the PET imaging performance. The desired improvements, initially focused on applications to proton therapy [103], could be achieved by increasing the thickness of the scattering layer and decreasing the relative distances between the scatterer and absorber rings (Figure 9D). In the framework of BARB, these efforts will be tailored to RIB and benefit from the reduced fluence of heavy ions compared to protons at the same treatment dose, resulting in relaxed signal processing rate requirements [104]. Moreover, higher resolution detectors tailored to small animal imaging will likely be employed, as currently under development in a joint effort between LMU and NIRS for a novel small animal in-beam PET scanner being realized for the SIRMIO ERC Consolidator Grant [30]. All these optimization design studies largely benefited from the collaboration between LMU and the University of Berkeley, USA (BACATEC; http://www.bacatec.de/) which aimed at developing a powerful simulation and image reconstruction framework, including a machine-learning algorithm for correct identification of the different types of event in the detectors [105]. Construction and detector testing for BARB will be performed in close collaboration between LMU and GSI groups.

## Conclusions

For many years, RIB have been proposed as the ideal bullet for image-guided particle therapy. The main problem has been the production of RIB and the low beam intensity. Research in this field started already at LBL and is currently mostly driven by NIRS in Japan, with interesting results and design of innovative PET detectors. These problems can be overcome by future, high-intensity accelerators, or by injection of RIB in conventional synchrotrons. NIRS and CERN are studying RIB sources that can work with current medical synchrotrons. The practical advantage of RIB therapy compared to conventional stable-ion treatments remains, however, not demonstrated. This is the goal of the BARB project, currently ongoing at FAIR in collaboration with LMU in Germany. BARB will exploit the intensity upgrade in FAIR-phase-0 and a novel γ-PET detector for beam visualization. BARB, NIRS, and CERN results in the coming decade will clarify whether there is a role of RIB in cancer treatment.

## Figures and Tables

**Table 1:** Positron-emitting isotopes that are found in proton therapy by target fragmentation and/or that have been considered as projectiles for RIB therapy.

Stable isotope	Positron-emitting isotopes	Half-life
^12^C	^11^C	20.33 min
	^10^C	19.3 s
^14^N	^13^N	9.97 min
	^12^N	11.0 ms
^16^O	^15^O	2.04 min
	^14^O	1.17 min
^19^F	^18^F	1.83 h
	^17^F	1.07 min
^20^Ne	^19^Ne	17.26 s
	^18^Ne	1.66 s
^31^P	^30^P	2.50 min
	^29^P	4.14 s

**Table 2:** A MOCADI simulation of the RIB intensity at GSI FRS.

Primary beam	Intensity at SIS-18 (per cycle)	Secondary beams	Production cross-section (mb)	Intensity at FRS (pps)	Energy (MeV/n)	Range in water (cm)	Range straggling (cm)
^12^C	8 × 10^10^	^10^C	4.8	2.3 × 10^7^	334	17.2	0.4
		^11^C	45.4	4.9 × 10^8^	347	20.1	0.5
^16^O	1 × 10^11^	^14^O	4.6	5.7 × 10^7^	405	18.4	0.4
		^15^O	45.6	9.1 × 10^8^	416	20.6	0.4
